# Is the EQ-5D fit for purpose in asthma? Acceptability and content validity from the patient perspective

**DOI:** 10.1186/s12955-018-0970-3

**Published:** 2018-08-03

**Authors:** Diane Whalley, Gary Globe, Rebecca Crawford, Lynda Doward, Eskinder Tafesse, John Brazier, David Price

**Affiliations:** 1 RTI Health Solutions, Manchester, UK; 2 Amgen Inc., Thousand Oaks, CA 91320 USA; 3 AstraZeneca Pharmaceuticals, Gaithersburg, MD USA; 4 School of Health and Related Research, University of Sheffield, Sheffield, UK; 5 Division of Applied Health Sciences, University of Aberdeen, Aberdeen, UK

**Keywords:** Asthma, Content validity, EQ-5D, Health state utility, Patient-reported outcomes, Qualitative

## Abstract

**Background:**

The increasing emphasis on patient-reported outcomes in health care decision making has prompted greater rigor in the evidence to support the instruments used. Acceptability and content validity are important properties of any measure to ensure it assesses the relevant aspects of the target concept. The purpose of this study was to evaluate the acceptability and content validity of the EQ-5D 5-Level (EQ-5D-5L) to assess the impact of asthma on patients’ lives.

**Methods:**

Qualitative interviews were conducted with 40 adults with asthma in the United Kingdom. The first 25 interviews used cognitive-debriefing methods to assess the relevance and acceptability of the EQ-5D-5L and two asthma-specific measures for comparison: an asthma-specific, preference-based measure (the Asthma Quality of Life Utility Index–5 Dimensions) and an Asthma Symptom Diary. The final 15 interviews combined concept elicitation to identify patient-perceived asthma impact, and cognitive debriefing to assess relevance and acceptability of the EQ-5D-5L and the Asthma Symptom Diary. Cognitive-debriefing feedback on the content of the measures was collated and summarized descriptively. The concept-elicitation data were analyzed thematically.

**Results:**

Participants were aged 20 to 57 years and 62.5% were female. Although some participants expressed positive opinions on aspects of the EQ-5D-5L, only the usual activities dimension was consistently considered relevant to participants’ asthma experiences. The mobility and self-care dimensions prompted strong negative reactions from some participants. Variations in interpretation of the mobility dimension and difficulties with multiple concepts in the pain/discomfort and anxiety/depression dimensions also were noted. Concepts reported by participants as missing included environmental triggers, asthma symptoms, emotions, and sleep. The EQ-5D-5L was the least preferred measure to describe the impact of asthma on participants’ lives. Participants reported shortness of breath and impact on activities as especially salient issues.

**Conclusions:**

The content of the EQ-5D-5L was poorly aligned with the patient-perceived impact of asthma, and the measure failed to meet basic standards for acceptability and content validity as a measure to assess the impact of asthma from the patient perspective. The shortcomings identified raise concerns regarding the appropriateness of the EQ-5D in asthma and further evaluation is warranted.

## Background

In the context of health economic evaluation, preference-based measures (PBMs) are used to represent the quality-of-life impact component of the quality-adjusted life-year (QALY) in cost-utility analyses [1]. The EQ-5D [2] is a patient-reported outcome (PRO) measure and is one of the most widely used PBMs for cost-utility analysis [3, 4]. The instrument is the measure of choice for many health technology assessment bodies, including the National Institute of Health and Care Excellence (NICE) in England [5], and is increasingly being used in the United States [6]. Although the EQ-5D is widely used, shortcomings have been noted in relation to its content coverage and its sensitivity and responsiveness in certain populations, particularly for patients with “mild” conditions [7, 8]. While studies have indicated that the increase in the number of response levels in the descriptive system of the 5-level EQ-5D (EQ-5D-5L) [7] has improved measurement precision [9] (although the currently unresolved discrepancies between the 3-level EQ-5D (EQ-5D-3L) and EQ-5D-5L value sets in the United Kingdom/England are noted [10, 11]), the EQ-5D-5L will not overcome any issues that are associated with irrelevant or missing content.

Concerns about the relevance and sensitivity of generic PBMs in some conditions has prompted the development of a number of condition-specific PBMs, including the Asthma Quality of Life Utility Index–5 Dimensions (AQL-5D) in asthma [12, 13], the EORTC-8D in cancer [14], and the NEWQOL-6D in epilepsy [15]. However, evidence for the increased sensitivity of such measures compared to generic PBMs has varied; for example, Lorgelly et al. [16] found similar levels of sensitivity in the EORTC-8D and the EQ-5D-3L in cancer, whereas McTaggart-Cowan et al. [17] demonstrated that the AQL-5D was better able to distinguish between differing levels of asthma control compared to three different generic instruments (Health Utilities Index-Mark 3, EQ-5D-3L, and the SF-6D). In relation to the NEWQOL-6D in epilepsy, Mulhern et al. [18] reported that, although the condition-specific PBM was generally more sensitive than the EQ-5D-3L, this did not result in large differences in utility.

Even if greater sensitivity can be demonstrated, the implementation of condition-specific PBMs for cost-utility analysis has been limited by the lack of ability to compare utility values across diseases and the potential of some condition-specific measures (although not all) to miss the impact of side effects and comorbidities [19]. Moreover, while it is recognized that generic PBMs sometimes may miss or underestimate important health-related quality of life (HRQOL) changes, from the perspective of economic evaluation, the focus is whether the measure “is sensitive enough” [16]. Given the lack of definitive criteria for sufficient sensitivity, this question is not easily answered. Furthermore, there is no consensus on the most appropriate way to assess this property for PBMs; Brazier and Deverill [20] suggested that traditional psychometric methods for testing construct validity and responsiveness often were not applicable to PBMs.

The increasing emphasis on PROs in health care decision making has prompted greater rigor in the evidence to support the instruments used [21, 22]. Any PRO instrument must be shown to be fit for purpose, given the intended context of use, and there are numerous guidelines outlining measurement quality standards (see for example, [23, 24]).

While there is debate on the applicability of some psychometric criteria to PBMs [20], content validity is a key requirement for any PRO measure, including PBMs, to ensure the instrument assesses the relevant and important aspects of the target concept of measurement [20, 25]. In the context of PBMs used to calculate QALYs, the target concept is HRQOL [5]. Although a consensus definition of HRQOL does not exist [26, 27], it is typically considered to be a multidimensional concept that encompasses domains relating to physical, mental, emotional, and social functioning [28]. Although NICE prioritizes generic instruments (and thus generic HRQOL) in its reference case, it recognizes the importance of understanding the patient perspective on the relevance of such instruments to the specific disease under consideration [5].

The purpose of this study was to explore the acceptability and content validity of the EQ-5D-5L from the perspective of patients with asthma. Asthma is an episodic condition, characterized by periods of disease control that are punctuated by debilitating, and potentially life-threatening exacerbations of varying durations. Questions have been raised about the ability of the EQ-5D to reflect the full impact of episodic conditions due its recall of “today” [29] and whether the measure is able to capture the impact of exacerbations in between attacks (e.g., fear of future attacks) [30]. Although studies have evaluated the content validity of the EQ-5D (see, for example, Matza et al. [31] and van Leeuwen et al. [32]), we are not aware of any studies that have evaluated this property directly with patients in asthma. Thus, this study sought to assess the acceptability and content validity of the EQ-5D-5L as a measure to assess the impact of asthma from the patients’ perspective.

## Methods

### Study design

A total of 40 qualitative interviews were conducted with adults with asthma. In the first 25 interviews (interview sample 1), cognitive debriefing was used to elicit feedback on the content of the EQ-5D-5L, as well as two asthma-specific measures for comparison purposes: the Asthma Symptom Diary (ASD) [33, 34], and the AQL-5D [12, 13]. Cognitive debriefing through qualitative patient interviews is used to establish the acceptability and content validity of PRO instruments [25, 35] by evaluating how patients interpret questionnaire items and confirming appropriateness, comprehensiveness, and understandability [25, 36]. The AQL-5D was selected as it is a frequently used as a condition-specific PBM in asthma. The ASD also was included to evaluate the acceptability to patients of a symptom-related impact measure; it is noted that, although criticized in the context of QALYs by some authors [13], symptom-based utility measures in asthma have been developed [37].

In the final 15 interviews (interview sample 2), the opportunity was taken to identify the concepts of relevance and importance to patients prior to cognitive debriefing of the instruments. Employing focused, open-ended questions relating to a specific topic of interest, concept elicitation is commonly used to elicit patients’ spontaneous self-reports of their experiences with their condition [38] and is a key method to establish content validity of PRO instruments [36]. To avoid overburdening participants, cognitive debriefing of the AQL-5D was not included in these 15 interviews.

The study was reviewed and granted approval from one of RTI International’s institutional review boards.

### Study sample

The study sample was a convenience sample of 40 adults with asthma recruited from the northwest region of the United Kingdom through a medical fieldwork agency. Participants were included if they were aged 18 years or older, had a self-reported physician-diagnosis of asthma, used at least one controller asthma medication, were able to read and complete an English-language paper questionnaire, and were able to provide written informed consent. Participants were excluded if they were aged over 50 years and had a history of smoking for 15 years or longer, or had a significant comorbidity. To minimize potential sampling bias arising from the convenience sampling approach, participants were recruited to represent a range of asthma severity, asthma control, and exacerbation history.

There are no definitive guidelines for determining sample sizes for qualitative research. In the context of cognitive-debriefing interviews, where the aim is to identify potential problems with a measure, sample sizes of between 5 and 15 are typical [39], although samples as high as 30 also have been recommended [40]. For concept elicitation, the emphasis is often on achieving concept saturation, that is, the point at which no new relevant information is elicited from subsequent interviews [36]. It has been suggested that for studies in which the aim is to understand perceptions and experiences in a relatively homogeneous group, as few as 12 interviews should be sufficient to reach saturation [41]. Thus, it was anticipated that the sample sizes of 40 for cognitive debriefing and 15 for concept elicitation would be adequate to achieve the objectives of this study.

### Study instruments

The EQ-5D [2] is a generic measure that was developed to assess health status across diseases on a common scale. The EQ-5D-5L [7] is a descriptive system comprising five dimensions (mobility, self-care, usual activities, pain/discomfort, and anxiety/depression), each with five response levels, ranging from no problems (1) to extreme problems (5). The dimensions for the EQ-5D were identified and refined through a detailed review of other available generic health measures and further empirical testing [42].

The ASD [33, 34] is a diary instrument designed to assess asthma symptoms and symptom-related impacts. The diary is intended to be completed by patients twice daily (morning and evening). The morning diary assesses the nighttime severity of four symptoms (wheezing, shortness of breath, cough, and chest tightness), as well as the number of nighttime awakenings. The evening diary assesses the same four symptoms and the extent to which activities were limited during the day. The content of the ASD was developed and refined based on clinical input and qualitative interviews with patients with asthma.

The AQL-5D [12, 13] is a PBM derived from the Asthma Quality of Life Questionnaire (AQLQ). The AQLQ [43] is a 32-item asthma-specific HRQOL measure comprising four domains: activity limitations, emotional function, exposure to environmental stimuli, and symptoms. The content of the AQLQ was developed and refined based on a literature review, existing HRQOL measures, discussions with physicians, and interviews and a survey with patients with asthma. The AQL-5D has five dimensions that were identified through principal components analysis of the AQLQ items: general symptoms (shortness of breath), sleep symptoms (interference with getting a good night’s sleep), activity (limitation with all activities done), emotion (concerns about having asthma), and environmental stimuli (experience of symptoms as a result of air pollution and weather).

Participants also completed questions on sociodemographics and asthma status. To assess asthma control, participants completed four questions outlined by the Global Initiative for Asthma [44], as well as the 6-item Asthma Control Questionnaire (ACQ-6) [45]. Scores on the ACQ-6 range from 0 to 6; higher scores reflect poorer asthma control. A score of 1.0 on the Asthma Control Questionnaire has been reported as the cross-over between well-controlled and not well-controlled asthma [46].

### Interview procedures

The interviews were conducted by experienced interviewers and were facilitated by an interview guide. Informed consent was obtained prior to initiating the interview and participants then completed the background and asthma control questions.

Interview sample 1 (*n* = 25) completed the EQ-5D-5L, the ASD, and the full AQLQ. The ordering of the instruments was varied to minimize bias. After completion of all three instruments, participants were asked debriefing questions to explore the acceptability, relevance, and comprehensiveness (i.e., whether any issues of importance were missing) of each instrument. For the AQLQ, participants were asked to consider only the five AQL-5D items during the debriefing. Participants rated each instrument on a scale from 1 (not relevant at all) to 10 (extremely relevant) in terms of its relevance to describe the effect that asthma has on their lives. Finally, participants were asked to select which instrument and which individual items (with no limit on how many items were selected) best described the effect that asthma has on their lives.

Participants in interview sample 2 (*n* = 15) were first asked about their experiences with asthma (including symptoms and asthma attacks) and the impact of asthma on daily life. Participants then completed the EQ-5D-5L and the ASD, and were asked debriefing questions and selected which instrument and which individual items best described the effect that asthma has on their lives. The ordering of instruments was alternated to minimize bias.

All interviews were audio recorded and detailed field notes were taken. The audio recordings from the second set of 15 interviews also were transcribed verbatim by a medical transcriptionist independent to the research team, to facilitate analysis of the concept-elicitation data.

### Analysis

Participants’ feedback on the EQ-5D-5L, the AQL-5D, and the ASD was collated and summarized using the interview field notes or transcript data as available and supplemented by the audio recordings, if needed. The concept-elicitation transcript data from interview sample 2 were analyzed thematically using Atlas.ti 7 coding software (Atlas.ti; Berlin, Germany). An initial coding frame was applied iteratively to the transcript data and was updated as themes were refined and new codes were developed. The analysis was conducted by two researchers: one researcher undertook the primary summarization or coding, and the second researcher read the field notes and/or transcripts and reviewed the summaries and applied codes. Any discrepancies were resolved by the two researchers.

The output from the qualitative analysis was a descriptive summary of participants’ feedback on the questionnaires and of the issues discussed in relation to the impact of asthma. Concept saturation in the concept-elicitation data was assessed by the emergence of new analysis codes across successive sets of three interview transcripts [36].

Participants’ ratings of the relevance of each instrument were summarized by the mean rating. Participants’ selections of the most relevant instrument and individual items to describe the effect that asthma has on their lives were summarized by frequency counts.

## Results

### Sample characteristics

The 40 participants ranged in age from 20 to 57 years, and 62.5% were female (Table 1). Although most participants (92.5%) reported their asthma to be either mild or moderate at the time of the interview, 85.0% were using two or more controller medications, 57.5% had uncontrolled asthma, and the mean ACQ-6 score (1.7) indicated not well-controlled asthma (Table 2).

**Table 1:** Demographic Characteristics of the Interview Sample

Characteristic	Interview Sample		Total Sample (*N* = 40)
	Sample 1 (*n* = 25)	Sample 2 (*n* = 15)	
Age (years)			
n	25	15	40
Mean (SD)	38.7 (11.1)	36.5 (10.1)	37.9 (10.7)
Median (Q1, Q3)	37 (32.0, 45.0)	34 (31.0, 40.0)	37 (31.3, 45.0)
Range	21–57	20–55	20–57
Sex, n (%)			
Male	10 (40.0)	5 (33.3)	15 (37.5)
Female	15 (60.0)	10 (66.7)	25 (62.5)
Relationships status, n (%)			
Married or living as married	17 (68.0)	8 (53.3)	25 (62.5)
Divorced	1 (4.0)	1 (6.7)	2 (5.0)
Single	7 (28.0)	6 (40.0)	13 (32.5)
Employment status, n (%)			
Working full time	13 (52.0)	8 (53.3)	21 (52.5)
Working part time	9 (36.0)	4 (26.7)	13 (32.5)
Retired	1 (4.0)	0 (0.0)	1 (2.5)
Student	1 (4.0)	2 (13.3)	3 (7.5)
Other	1 (4.0)	1 (6.7)	2 (5.0)

*Q* quartile*, SD* standard deviation

**Table 2:** Asthma Status of the Interview Sample

Characteristic	Interview Sample		Total Sample (*N* = 40)
	Sample 1 (*n* = 25)	Sample 2 (*n* = 15)	
Duration of asthma diagnosis (years)			
n	24	15	39
Mean (SD)	24.3 (12.4)	22.2 (10.4)	23.5 (11.6)
Median (Q1, Q3)	25.5 (13.0, 32.0)	20.0 (15.0, 30.5)	25.0 (13.5, 31.0)
Range	3–57	2.5–40	2.5–57
Self-reported severity of asthma, n (%)			
Mild	8 (32.0)	3 (20.0)	11 (27.5)
Moderate	15 (60.0)	11 (73.3)	26 (65.0)
Severe	2 (8.0)	0 (0.0)	2 (5.0)
Very severe	0 (0.0)	1 (6.7)	1 (2.5)
GINA asthma control,^a^ n (%)			
Well controlled	1 (4.0)	1 (6.7)	2 (5.0)
Partly controlled	10 (40.0)	5 (33.3)	15 (37.5)
Uncontrolled	14 (56.0)	9 (60.0)	23 (57.5)
ACQ-6 score			
n	25	15	40
Mean (SD)	1.6 (0.9)	1.9 (0.7)	1.7 (0.8)
Median (Q1, Q3)	1.7 (0.8, 2.0)	1.8 (1.6, 2.3)	1.8 (1.2, 2.3)
Range	0.3–3.7	0.7–3.3	0.3–3.7
Number of controller medications, n (%)			
1 controller	5 (20.0)	1 (6.7)	6 (15.0)
2 controllers	18 (72.0)	10 (66.7)	28 (70.0)
3 controllers	2 (8.0)	4 (26.7)	6 (15.0)
Number of attacks in last 2 years, n (%)			
0	9 (36.0)	1 (6.7)	10 (25.0)
1	5 (20.0)	5 (33.3)	10 (25.0)
2	6 (24.0)	7 (46.7)	13 (32.5)
3 or more	5 (20.0)	2 (13.3)	7 (17.5)

*ACQ-6* 6-item Asthma Control Questionnaire, *GINA* Global Initiative for Asthma, *Q* quartile, *SD* standard deviation

^a^GINA asthma control was based on participants’ responses to four questions on activity limitations, daytime symptoms, night awakening, and medication use: well controlled = “no” responses to all four questions; partly controlled = “yes” responses to one or two questions; and uncontrolled = “yes” responses to three or four questions [44]

### Evaluation of the EQ-5D-5L

Participants in both samples had diverse opinions on the relevance of the EQ-5D-5L. Although some participants described it as measuring the issues impacted by asthma, other participants considered it to be too general and stated that some questions were not relevant to their experiences of asthma. A number of participants indicated that some of their responses would be unlikely to change even when their asthma was at its most severe.

Individual participants found some of the questions, particularly mobility and self-care, to be offensive. Such participants spoke with indignation about being asked these questions and dismissed them as being related to conditions that were more physically debilitating, such as arthritis:

“Some of them are almost a bit insulting…They remind me of the questions we had when Mum was going in a home and we were getting a statement to assess and…you know. When I read those, it was like, ‘What? No!’ Disgrace.” (37-year-old female)

#### Mobility

Many participants (*n* = 17) did not consider that the mobility dimension of the EQ-5D-5L related to their experiences with asthma:

“Mobility isn’t relevant because I don’t see that it does—well, it doesn’t affect my personal experience of asthma because I’ve never had a problem with mobility over it” (41-year-old male)

The level of functional impairment reflected in the dimension was reported as being relevant only during periods of severe illness or during an asthma attack. Participants differed in their interpretation of the dimension as including movements such as climbing stairs or walking uphill. Such differing interpretations could affect participants’ responses; for example, one participant answered ‘slight problems’ to the question, but when probed, she stated that she had severe problems walking up an incline.

#### Self-care

Self-care was not a relevant issue for a majority of participants (*n* = 29), either in living with asthma day to day or during an attack. Individual participants remarked that self-care could possibly become an issue if they were to ever become very ill.

“… personally from my asthma experience I find the washing and dressing myself question incredibly strange…Because I have never ever struggled to wash or dress myself.” (20-year-old female)

#### Usual activities

The usual activities item was considered the most important of the five dimensions in the EQ-5D-5L. For nearly all participants (*n* = 37), this dimension was a relevant and central issue in their asthma:

“I think we’re talking here about activities of leisure and family. I would say I have slight problems for the reasons I’ve talked about really, energy levels and fitness, which are affected by the asthma.” (41-year-old male)

“Yes, the ‘usual activities’ is relevant all the time because that is the rollercoaster thing, isn’t it?” (30-year-old female)

#### Pain or discomfort

Individual participants described asthma as painful and thus welcomed the inclusion of the pain or discomfort item in the EQ-5D-5L:

“It’s [asthma’s] really painful, and it just doesn’t seem to be one of the things that is ever factored into it, so it’s actually quite nice to see that there” (33-year-old female).

However, for most participants (*n* = 19), discomfort was a more relevant concept than pain; and the combination of pain and discomfort in a single question had implications for how participants responded. For example, some participants who experienced discomfort in relation to asthma rated their level of ‘pain or discomfort’ as less severe because they did not have pain. In contrast, other participants answered only in relation to discomfort because pain was irrelevant.

“… they [pain and discomfort] are two different words. I don’t think they should be allowed to be joined together...I have never described that [asthma] as painful. Discomfort, a little uncomfortable sometimes, but never painful. It is not the word for an asthma attack. I don’t think I have experienced it anyway...with the pain there I would be tempted just to tick the first one.” (26-year-old male)

#### Anxiety or depression

Although neither anxiety nor depression was particularly relevant for many (*n* = 20) participants, anxiety generally was considered to be the more relevant issue. Four participants suggested that feelings such as concern, frustration, or embarrassment were more suitable expressions of the psychological impact of asthma. In addition, anxiety and depression were considered separate issues and their combination in a single item was problematic. The implication of clinical depression provoked strong reactions from individual participants:

“I just don’t like it being linked to the depression bit though, so I would say that I’m anxious about it; but when they put depression next to it as well, I’m not depressed so I’d say it’s two separate questions…I just don’t like those two; they’re really not nice phrases.” (30-year-old female).

### Evaluation of the EQ-5D-5L compared with the ASD and the AQL-5D

In interview sample 1, the 1-to-10 rating for relevance was highest (most relevant) for the ASD (mean: 8.5), followed by the AQL-5D (mean: 7.5) and the EQ-5D-5L (mean: 5.6). In the head-to-head comparison, more participants selected the AQL-5D (*n* = 12) as the best instrument to describe the effects of asthma on their lives, compared with the ASD (*n* = 8) or the EQ-5D-5L (*n* = 2). The remaining three participants were undecided between the ASD and AQL-5D. In a similar head-to-head comparison of the EQ-5D-5L and the ASD in interview sample 2, the majority (*n* = 10) preferred the ASD, four participants preferred the EQ-5D-5L, and one participant was undecided.

Across both sets of interviews, the reason given for preferring the EQ-5D-5L often related to it assessing impact, rather than just the cause of the impact (i.e., symptoms):

“Well because it’s [EQ-5D-5L’s] not just traditional symptoms. I think the other questionnaire is about symptoms. It’s like, can you breathe? Are you coughing a lot? Are you waking up? Can you do stuff? But this one’s, it’s almost like the next level to that. It’s almost like the impact it has on your life rather than the impact it has on your body.” (33-year-old female)

However, many (*n* = 15) of the participants who preferred either the AQL-5D or the ASD commented that the EQ-5D-5L dimensions lacked relevance to the impact of asthma:

“… these [EQ-5D-5L questions] are so outside my normal sphere of experience of asthma that I can’t really relate to them too much.” (34-year-old male)

**Fig. 1:**
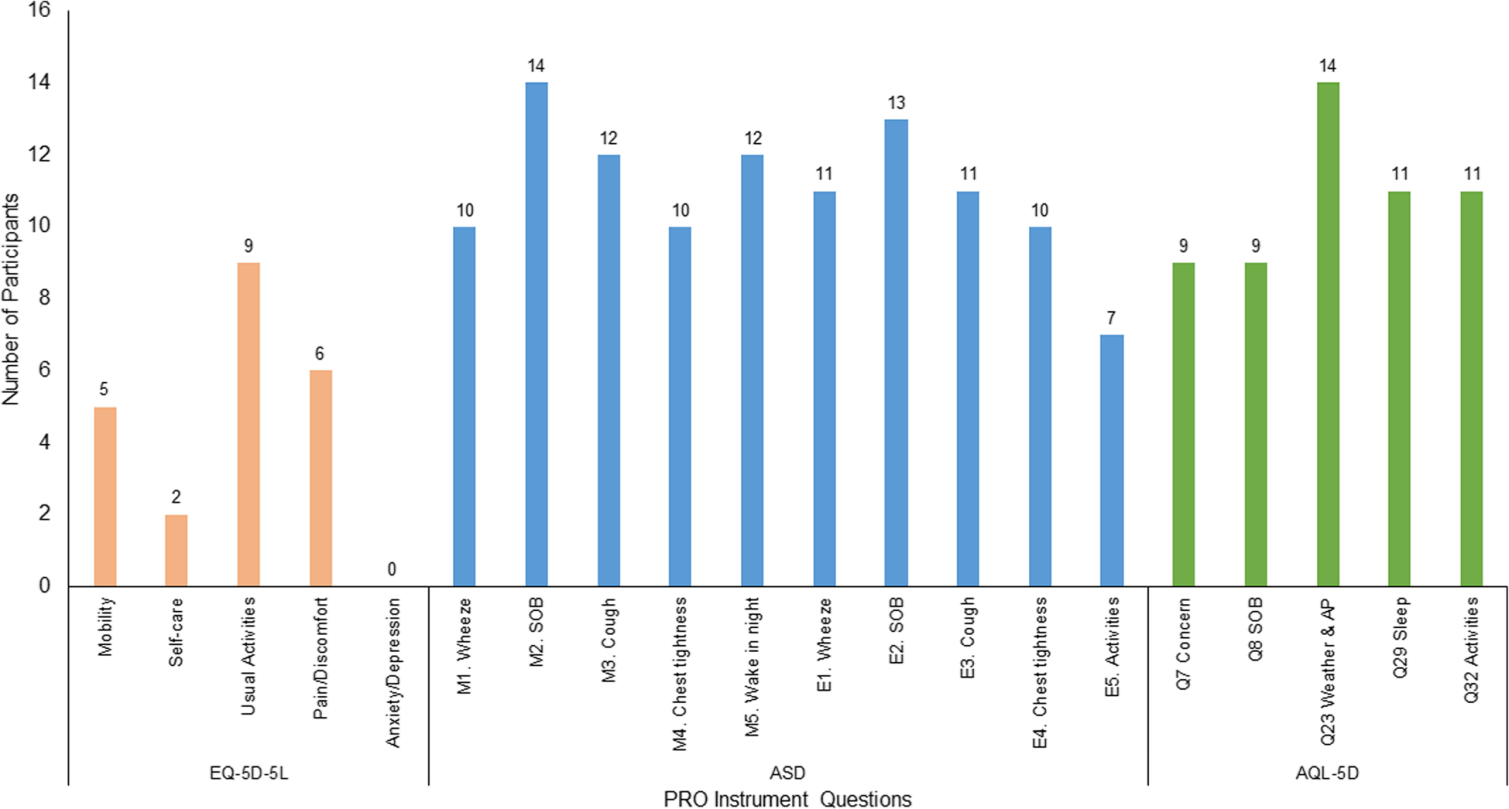
Selection of Best Questions (Interview Sample 1; *n* = 25). *AP* air pollution; *AQL-5D* Asthma Quality of Life Utility Index–5 Dimensions; *ASD* Asthma Symptom Diary; *EQ-5D-5L* EQ-5D 5 Level; *PRO* patient-reported outcome; *SOB* shortness of breath. Note: Participants were able to select more than one item

In interview sample 1, the individual items chosen most frequently to best describe the impact of asthma were the symptom and night awakening items from the ASD and the weather and air pollution, sleep, and activities items from the AQL-5D (Fig. 1). The EQ-5D-5L self-care item was selected by only two participants, and the anxiety/depression item was not chosen at all.

**Fig. 2:**
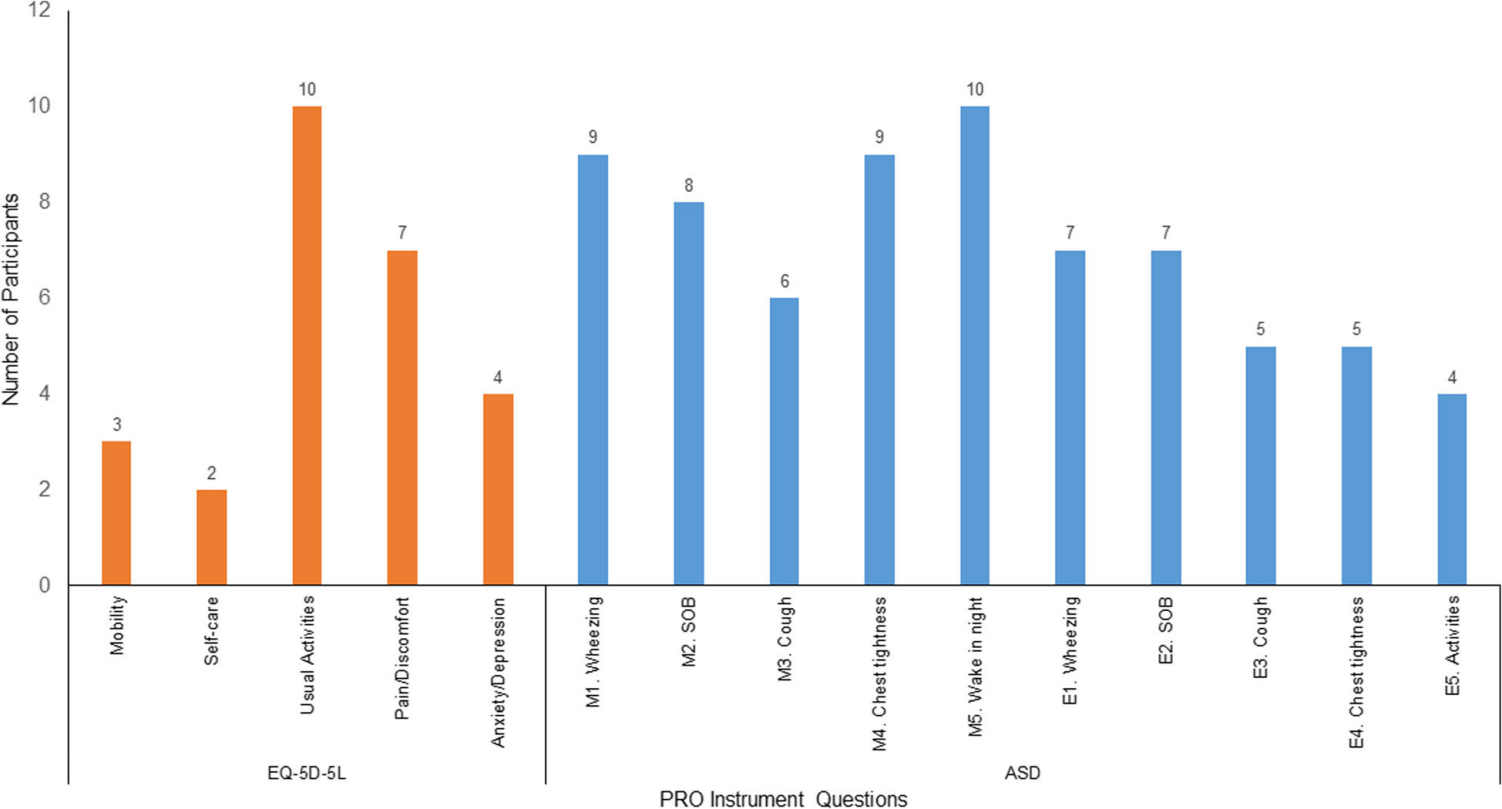
Selection of Best Questions (Interview Sample 2; *n* = 15). *ASD* Asthma Symptom Diary; *EQ-5D-5L* EQ-5D 5 Level; *PRO* patient-reported outcome; *SOB* shortness of breath. Note: Participants were able to select more than one item

In interview sample 2, the questions selected the most often were the symptom (except cough) and night awakening items of the ASD and the usual activities and pain/discomfort dimensions of the EQ-5D-5L (Fig. 2). Once again, the mobility and self-care items of the EQ-5D-5L were selected by relatively few participants.

Across both interview samples, the issues identified as being missing from the instruments included environmental triggers, asthma symptoms, emotions (other than anxiety or depression), and sleep for the EQ-5D-5L; environmental triggers, pain and fatigue, emotions, and medication use for the ASD; and environmental triggers and wheeze for the AQL-5D.

### Areas of impact of asthma

**Fig. 3:**
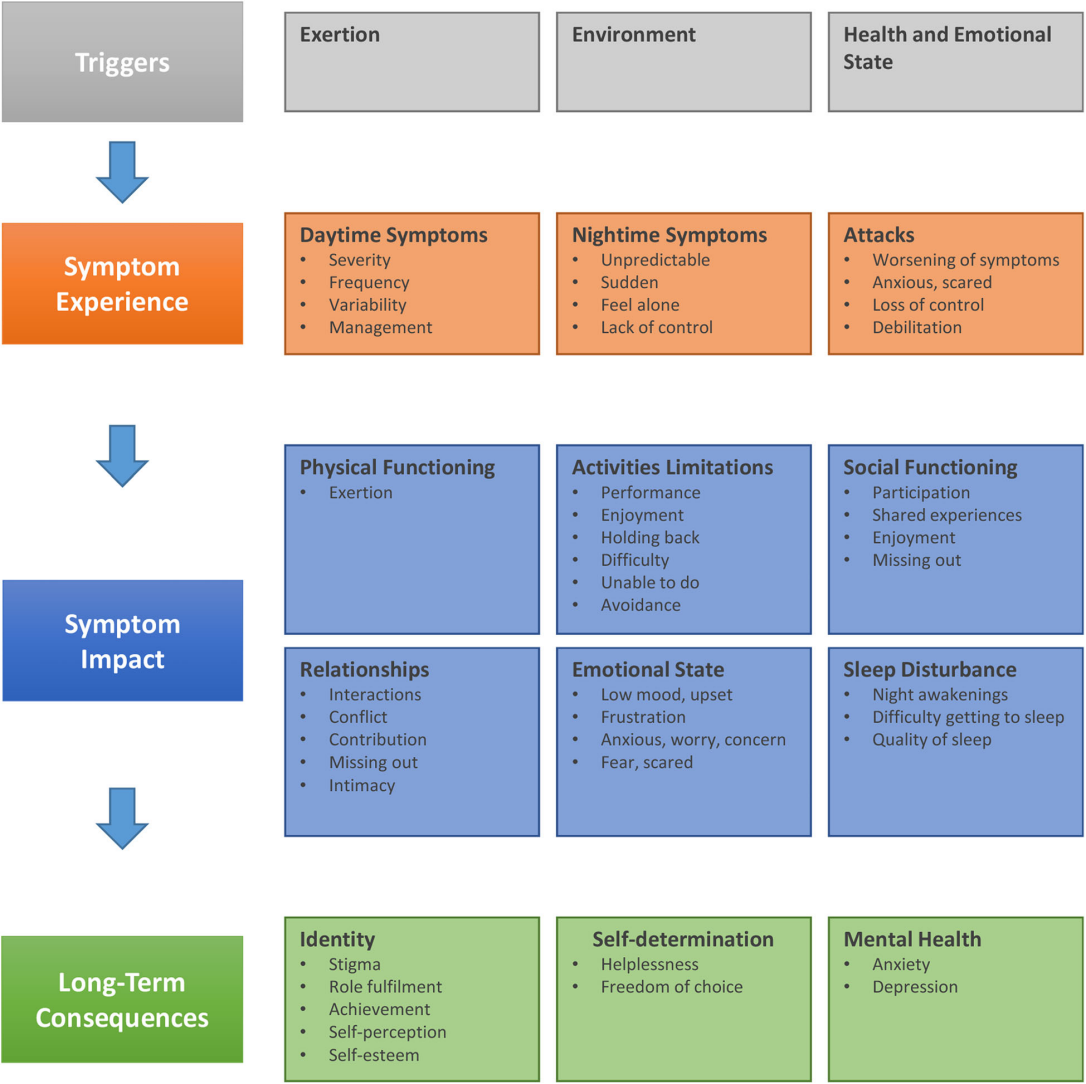
Asthma Impact Concept Map

Figure 3 presents the key concepts identified from the interview sample 2 concept-elicitation data; 79 impact areas across 15 key concepts were identified. Over 90% of the impact areas were identified in the first nine interviews, providing evidence of concept saturation.

Shortness of breath was most frequently reported as the most bothersome symptom. Nighttime symptoms had particular significance for a number of participants; participants described feeling more frightened, isolated, and helpless with their nighttime symptoms. Asthma attacks, particularly severe attacks, were all-consuming experiences, during which participants felt frightened, anxious, panicked, embarrassed, helpless, vulnerable, and out of control. At such times, attention tended to focus on symptoms, and the immediate aftermath was associated with relief, embarrassment, and being physically drained. In between attacks, participants described feeling worried and concerned about having another attack and being alone when an attack occurred.

Activity limitations was the most significant impact of asthma, particularly exercise and taking part in physical activities with family and friends. Participants described periods of time when they were unable to be (or avoided being) physically active; for some participants, prolonged periods of inactivity had a negative impact on their feelings of health and well-being.

“Because I’ve been sick, I can’t exercise, I can’t lose weight, I get fatter, and then can’t breathe and then can’t exercise” (37-year-old female)

Participants’ ability to participate or engage socially was affected as a result of their symptoms or through the avoidance of triggers.

“… it is the epitome of having like friends, you not being able to do stuff with them, and, again, it's not like a massive chunk of my life, but it is a chunk of my life” (33-year-old-male)

Some participants described their asthma as being part of their everyday life; such individuals accepted their limitations and adjusted their lives accordingly.

“It’s just one of those things. It’s frustrating, but I have found ways around it. I mean, I will never be able to play at that level of semi-professional sport purely because of my respiratory endurance and things like that, but I can still, you know, I can still know when and where my limit is. I can still enjoy exercise to a point, just not really competitive exercise” (20-year-old female)

For other participants, however, the limitations of asthma had affected their sense of achievement and feelings of self-worth.

“… so wheezing makes me less able to communicate effectively in a professional sense. It makes me feel less willing, or less—I suppose in myself it makes communication more difficult, because if you’re wheezing, you’re not able to speak and express yourself as well, so it is quite debilitating.” (41-year-old male)

“Just deeply frustrated because I just don’t feel I can reach my potential because of it. Whether it’s sports or relationships or work, I just don’t feel like those things are as fruitful as they could be” (25-year-old female)

Table 3 provides an overview of how the key concepts identified from the interviews related to the content of the EQ-5D-5L.

**Table 3:** Alignment of the EQ-5D-5L Dimensions to Key Impact Concepts

Concept	EQ-5D Dimension	Interview Findings
Physical functioning	Mobility and Usual activities	▪ Participants reported difficulties with physical functions that required respiratory effort (e.g., climbing stairs, walking uphill, and running). ▪ EQ-5D-5L usual activities dimension was generally acceptable to participants, and the dimension reflected the activity limitation concepts raised in the concept-elicitation interviews. ▪ The level of mobility reflected in the EQ-5D-5L mobility dimension (i.e., problems with walking about) was poorly aligned to the issues experienced by most of the participants.
Self-care	Self-care	▪ The EQ-5D-5L self-care item was almost universally considered to be neither relevant nor important to the patient experience of asthma.
Emotions	Anxiety or depression	▪ Participants reported a range of emotions in relation to their asthma, e.g., frustration, low mood, worry, and embarrassment. ▪ The expression of anxiety in the EQ-5D-5L was not completely aligned with the emotions expressed in the interviews, but the concept was seen as more relevant than depression. ▪ The combination of depression and anxiety in one dimension was unacceptable to some participants and led to inconsistencies in responses; participants answered variously in terms of one or both of the issues, despite the question and response options relating only to anxiety or depression. ▪ Participants identified emotions other than anxiety or depression as being missing from the EQ-5D-5L.
Asthma symptoms	Pain or discomfort	▪ The symptoms of asthma were central to the impact of asthma; this impact was expressed in terms of the experiential effect (e.g., the unpleasant and frightening experience of the symptoms themselves) and the impact on participants’ lives (e.g., being unable to take part in activities). ▪ Shortness of breath was described as the most bothersome symptom. ▪ Discomfort and pain were less commonly reported and were more distal to the asthma experience; discomfort was generally more relevant than pain. ▪ The combination of pain and discomfort in one dimension was unacceptable to some participants and led to inconsistencies in responses; participants answered variously in terms of one or both of the issues, despite the question and response options relating to pain or discomfort.
Nighttime symptoms and sleep disturbance	Not assessed	▪ Nighttime symptoms and the associated sleep disturbance were often reported by participants and had particular salience for some individuals. ▪ These issues were highlighted by some participants as missing from the EQ-5D-5L.
Social functioning	Not assessed	▪ For some participants, asthma had a considerable impact on their ability to go out or engage socially.
Relationships	Not assessed	▪ For individual participants, asthma impacted relationships with friends and family, often as a result of not being able to do or take part in certain activities.

*EQ-5D-5L* EQ-5D 5 Level

## Discussion

The findings of this qualitative study provide evidence of shortcomings in the EQ-5D-5L with respect to its acceptability and content validity in asthma. With the notable exception of the usual activities dimension, many of the participants considered the EQ-5D-5L dimensions to be partially or completely irrelevant, either because the concept was not relevant to their experiences with asthma or because the concept was not expressed in a relevant way. Some dimensions yielded inconsistent responses due to variability in interpretations of the level of impairment reflected in the dimension and/or because the dimension combined multiple concepts. These issues leave open the potential for individuals experiencing the same level of impact to give different responses and thus having different utility index scores, as these are derived from the dimension scores using preference-based utility weights. Although this could apply to any patient population, it is especially likely in asthma where only one of the multiple concepts in a given item is relevant, as was the case for many of our study’s participants. In the context of PRO instruments, items associated with such problems would be strong candidates for removal from a measure [47].

The findings in this study resonate with a qualitative evaluation of the EQ-5D-5L in patients with diabetes [31], in which the EQ-5D-5L items were reported as being relevant for between 24% (self-care) and 68% (anxiety/depression) of participants. Approximately one-half of the sample said that the overall instrument was relevant to their experience. The authors concluded that their findings raised questions about the content validity of the EQ-5D for diabetes [31]. Some participants indicated that while the EQ-5D-5L could be relevant to other and possibly more severe patients, it was not relevant to their own personal experience. Some patients in our study similarly commented that the EQ-5D would only be relevant when they were at their most severe (e.g., during an attack); it is noted that the patients were relatively severe in terms of asthma control and patient-reported exacerbation history and medications. Matza et al. [31] further noted that interviewees identified issues not captured in the EQ-5D-5L, for example, specific activities, comorbidities, diabetes symptoms, diabetes treatment, emotions other than anxiety or depression, dietary issues, relationships, and social life. For the current study, concepts reported as missing from the EQ-5D-5L included asthma triggers, asthma symptoms, emotions other than anxiety or depression, and sleep. Whether or not these concepts constitute important aspects of HRQOL is contingent on the definition of the concept. However, as outlined previously, there is no agreement in the literature on the definition of HRQOL [26, 27], and an in-depth analysis of the concept is beyond the scope of this paper. Nonetheless, from the patient perspective, these issues represented important areas of impact of asthma.

It is unsurprising that the EQ-5D-5L was viewed as less relevant than the two disease-specific instruments used in this study. Both the ASD and AQLQ were designed to focus on the issues of relevance to asthma and were developed using considerable patient input [33, 43]. Direct patient input is now deemed fundamental but the original EQ-5D-3L was developed at a time when qualitative work in PRO instrument development was neither a requirement nor commonly done. Thus, although the EuroQol Group discussed obtaining patient input via a survey, they decided instead to select the dimensions for inclusion based on a review of existing generic health measures [42]. Nonetheless, it is essential that the EQ-5D’s context of use is taken into account when evaluating its quality. As a generic instrument used for economic evaluation, the dimensions of the EQ-5D are intended to be general, both in concept and applicability, and would not be expected to be as proximal to the disease as a disease-specific measure used to assess outcome in clinical trials. In this respect, there is merit in the recommendation made by the Panel on Cost-Effectiveness in Health and Medicine in the United States that, although the use of generic PBMs for the reference case in cost-effectiveness analyses facilitates comparability across studies, there is value in presenting utility estimates obtained from other sources alongside the reference case [48].

Regardless of the context of use, a fundamental requirement for any PRO instrument is that it should not alienate patients; that is, even if a given question does not apply, it should still be acceptable to patients to complete. However, a notable minority of participants in this study expressed surprise, and at times reacted with indignation, in relation to the EQ-5D-5L mobility and self-care dimensions.

The 2013 NICE guideline requires sponsors to provide qualitative, empirical evidence to show that the EQ-5D is not appropriate for a given population [5] but neglects to provide guidance as to what level of failure constitutes ‘inappropriate,’ as noted by Matza et al. [31]. Such lack of clarity perpetuates poor PRO measurement practice in a context in which scientific rigor is crucial. From a regulatory standpoint, the problems with acceptability and content validity identified in this study would be sufficient to conclude that the EQ-5D-5L was not an acceptable PRO instrument [21]. NICE takes a different perspective, considering a lack of content validity as relevant only if supported by evidence that construct validity and responsiveness also are adversely impacted [5].

Although studies have demonstrated variability in the EQ-5D scores in patients with different levels of asthma control and some other known groups [30], ceiling effects have been noted in asthma [49], and studies have generally found the measure to be less sensitive than asthma-specific PRO instruments [30], including the asthma-specific preference-based AQL-5D [17, 50]. A systematic review concluded that the EQ-5D was less appropriate than other measures in patients with mild disease or good disease control [30]. Much of this evidence pertains to the EQ-5D-3L but the extent to which the EQ-5D-5L overcomes these shortcomings is unknown. However, such evidence concurs with the present study in which participants’ comments suggest that much of the content of the EQ-5D-5L would be capable of demonstrating the impact of asthma only at its most severe. Thus, the wider evidence suggests that the EQ-5D has limitations in asthma, and especially so in mild asthma. Under such circumstances, use of EQ-5D in health technology assessment decision models could undervalue the benefit of effective interventions.

The interviews were designed to explore the appropriateness of the instruments to describe the impact of asthma from the patients’ perspective. Although it was evident that some participants made a clear distinction between symptoms and their impact, it is likely that not all participants differentiated their asthma experience in this way. Thus, although some participants reported asthma symptoms as being key omissions from the EQ-5D-5L, this is not necessarily a threat to the instrument’s validity as a measure of health impact. However, it is noted that pain is measured by the EQ-5D-5L and is considered to be a symptom concept [26]. As another symptom, shortness of breath, would be equally valid but more relevant to assess in asthma than pain. Indeed, in an exploration of the potential for a respiratory EQ-5D “bolt-on,” shortness of breath was identified as an appropriate candidate for an additional dimension [51]. The bolt-on approach is in its infancy, and different additional dimensions have varied in terms of their impact on the EQ-5D-3L descriptive system [52–56]. The viability of incorporating shortness of breath into the EQ-5D, whether through a bolt-on or some other means, would need to be explored.

There were a number of limitations associated with this study. The convenience sampling approach meant that asthma status was determined through self-report, although participants presented their asthma medications at the time of interview. The sample size has implications for the generalizability of the findings to the wider asthma population. However, a sample of 40 is typical of qualitative studies and above that employed in similar studies [31, 57]. In the context of cognitive debriefing, the sample size was considered adequate to confirm understanding and identify any problems with the instrument items [39, 40].

It is possible that participants’ opinions of the EQ-5D-5L could have been influenced by the other questionnaires completed. Ordering effects were mitigated by varying the order in which the instruments were completed, and there were no substantial differences between the two samples in the opinions expressed. Knowing that they were taking part in an asthma study may have focused participants’ attention on asthma. However, EQ-5D data used in economic evaluations are often collected in a disease-specific context (e.g., an asthma clinical trial) and administered alongside disease-specific instruments. It also is possible that the participants highlighted aspects of the EQ-5D-5L that they would not notice when completing the measure in a clinical study. However, the other two instruments were subject to the same focusing effects, but did not attract the same strength of criticism.

## Conclusions

The issues identified in this study raise questions regarding the appropriateness of the EQ-5D-5L to assess outcomes in asthma. Although never intended to evaluate change before and after treatment, the EQ-5D-5L is often used in this way and we would argue that for this context, the EQ-5D-5 L is undoubtedly suboptimal in asthma. For the purpose of economic evaluation in asthma, the issues identified are sufficient to warrant further consideration of the suitability of the EQ-5D. If decision makers are to employ PRO measures in their deliberations, it is crucial that the instruments used meet at least the most basic scientific standards for acceptability and content validity. In the current study, the EQ-5D-5L was shown to fall short of these standards. Further empirical research is needed to justify the appropriateness of the EQ-5D-5L in asthma.

## Abbreviations

ACQ-6: 6-item Asthma Control Questionnaire
AP: air pollution
AQL-5D: Asthma Quality of Life Utility Index–5 Dimensions
AQLQ: Asthma Quality of Life Questionnaire
ASD: Asthma Symptom Diary
EQ-5D-3L: 3-level EQ-5D
EQ-5D-5L: 5-level EQ-5D
GINA: Global Initiative for Asthma
HRQOL: Health-related quality of life
NICE: National Institute of Health and Care Excellence
PBM: Preference-based measure
PRO: Patient-reported outcome
Q: Quartile
QALY: Quality-adjusted life-year
SD: Standard deviation
SOB: Shortness of breath
